# Molecular mechanism for impaired suppressive function of Tregs in autoimmune diseases: A summary of cell‐intrinsic and cell‐extrinsic factors

**DOI:** 10.1111/jcmm.15743

**Published:** 2020-09-02

**Authors:** Luting Yang, Gang Wang, Haibin Xia

**Affiliations:** ^1^ Laboratory of Gene Therapy Department of Biochemistry College of Life Sciences Shaanxi Normal University Xi’an China; ^2^ Department of Dermatology Xijing Hospital Fourth Military Medical University Xi’an China

**Keywords:** cell‐extrinsic factors, cell‐intrinsic factors, immunosuppression, molecular mechanism, regulatory T cells

## Abstract

Regulatory T (Treg) cells are responsible for maintaining immune homeostasis and preventing autoimmunity. In immune homeostasis condition, Tregs exert their suppressive function through inhibiting the proliferation of effector T cells. In response to environmental signals, Tregs display phenotypic heterogeneity and altered stability, which endows their suppressive function in a context‐dependent manner. Compelling evidence indicates deficiency of Treg suppressive function is related to the immunopathogenesis of various autoimmune diseases. Consequently, it is vital to further our understanding of the molecular mechanism accounting for the regulation of Treg suppressive functions. In this review, we outline the current knowledge that highlights how cell‐intrinsic factors, such as inflammatory cytokines, transcription factors, signalling pathways, post‐translational modification (PTM), miRNAs, protein and protein complex, and cell‐extrinsic factors orchestrate the suppressive function of Tregs. Improved understanding of the molecular mechanism related to the suppressive functional property of Tregs should provide new insights into autoimmunity and disease pathogenesis, which offers opportunity for identifying new therapeutic targets for Treg‐related autoimmune diseases and cancers.

## 1 INTRODUCTION

Regulatory T cells (Tregs) are a subset of CD4^+^ T cells which exerts immunosuppressive functions. Tregs are essential in maintaining the self‐tolerance and cell homeostasis in healthy individuals. Although surface molecules like CD25 and CD127, and immune checkpoint molecules such as CTLA4, GITR and LAG‐3 have been suggested as the markers of Tregs, accumulating evidences suggest that their expression are not Treg‐specific. For example, upon activation, CTLA‐4, GITR and LAG‐3 are expressed on all CD4^+^ T cells.^1^ Controversial opinions have been proposed on regarding Foxp3 as a marker of Tregs. Despite the fact that a population of Foxp3^+^CD4^+^T cells do not express CD25, Foxp3 expression is more abundant in CD4^+^CD25^+^Treg cells. The importance of role of Foxp3 in the development of Tregs has been noticed since mutation of Foxp3 has been found to lead to severe multiorgan autoimmune syndromes including XLAAD and IPEX.^2^ Moreover, Foxp3 expression is essential for the Treg signature and immunosuppressive function. Epigenetic modification and post‐translational modification of Foxp3 which reduced the expression of Foxp3 will impair the differentiation of Tregs and their suppressive function.^3^ Thus, Foxp3 is regarded as the master transcription factor of Tregs.

Dysregulation of Tregs has been related to the pathogenesis of various autoimmune disease, such as psoriasis, rheumatoid arthritis (RA), systemic lupus erythematosus (SLE) and diabetes.^4^ The impaired suppressive function of Tregs will lead to uncontrolled proliferation of effector T cells, which exaggerate the inflammatory process and contribute to disease pathogenesis. Therefore, it is of importance to illustrate the molecular mechanism accounting for dysfunctional Tregs. The present review summarizes various factors, including inflammatory cytokines, transcription factors, miRNAs, post‐translational modifications and extrinsic factors such as impact by other cells. We do not include metabolic factors and regulation of Foxp3 by epigenetic modification as there are already detailed reviews in this field. Our purpose is to briefly summarize the current knowledge on the factors that influence the suppressive functions of Tregs and to provide theoretical basis for directing studies in the field of Tregs in different disease.

## 2 MOLECULAR MECHANISM ACCOUNTING FOR IMPAIRED SUPPRESSIVE FUNCTION OF TREGS

### 2.1 Inflammatory cytokines

The microenvironment in patients with autoimmune diseases is very complex and encompasses a series of pro‐inflammatory cytokines such as IL‐6, TNF‐α, IL‐21 and IFN‐γ. Despite the fact that these pro‐inflammatory cytokines are critical in driving the plasticity of Tregs, they are capable of regulating the suppressive function of Tregs.

In vitro, IL‐6 addition induced a significant decrease in both the proportion of CD4^+^CD25^+^Foxp3^+^ Tregs and their suppressive functions.^5^ Moreover, in psoriasis, it is discovered that elevated IL‐6 from endothelial cells, dendritic cells and Th17 cells induced phosphorylation of STAT3 in both Tregs and Teff cells, which dampened the suppressive function of Tregs to Teff cells.^6^

IL‐21, a CD4^+^ T cell–derived cytokine, is shown to enhance inflammatory response in autoimmune disease. In one study, it is suggested that IL‐21 rendered CD4^+^CD25^‐^ T cells resistant to Treg‐mediated suppression, which impaired the suppressive function of Tregs.^7^ Other mechanisms relating to IL‐21‐mediated suppression of Foxp3 have been proposed. One possibility might be that IL‐21 could reduce the expression and stability of Foxp3 in CD4^+^ T cells.^8^ By using IL‐21R^‐/‐^ mice in asthma and colitis, Tortola et al^9^ uncovered a direct effect of IL‐21 on promoting apoptosis of Tregs. By further investigation, IL‐21 was shown to interfere with expression of Bcl‐2 family genes which sensitized these Tregs to apoptosis.

TNF‐α has also been shown to regulate the inhibitory function of Tregs. However, contradictory effects of TNF‐α on the function of Tregs have been reported. TNF‐α is long considered to be potent pro‐inflammatory cytokines that are implicated in the pathogenesis of various diseases; however, the pleiotropic effect of TNF‐α such as anti‐inflammatory function has been proposed. In rheumatoid arthritis (RA), TNF‐α decreased the expression of Foxp3, which down‐regulated the suppressive function of Tregs. Anti‐TNF therapy with infliximab restored the suppressive function of Tregs through a mechanism involving an increase in Foxp3.^10^ Foxp3 is the master transcription factor of Tregs, and various studies suggested that TNF‐α could modulate Foxp3 expression through an epigenetic or post‐translational modification. Nie et al^11^ discovered that TNF‐α down‐regulated Treg suppressive function via dephosphorylation of Foxp3. Urbano et al^12^ reviewed a tumour necrosis factor receptor 2 (TNFR2)–induced hypomethylation of Foxp3, which enhanced Treg stability and maintained suppressive function of Tregs. Besides, recent studies have showed the promotive effects of TNF‐α on the suppressive function of Tregs. This is evidenced by the fact that TNF‐deficient mice exhibited decreased immunosuppressive properties and developed exacerbated experimental autoimmune encephalomyelitis (EAE).^13^ In graft‐versus‐host disease and tumour environment, TNF‐α was shown to enhance the suppressive function of Tregs mainly through a TNF‐α‐TNFR2 signalling.^14^

Other pro‐inflammatory cytokines such as IL‐1β was also capable of abrogating the suppressive function of Tregs.^15^ Clarifying the complex crosstalk of these cytokines is critical to understand the mechanisms accounting for dysfunctional Tregs.

### 2.2 Transcription factors

**FIGURE 1:**
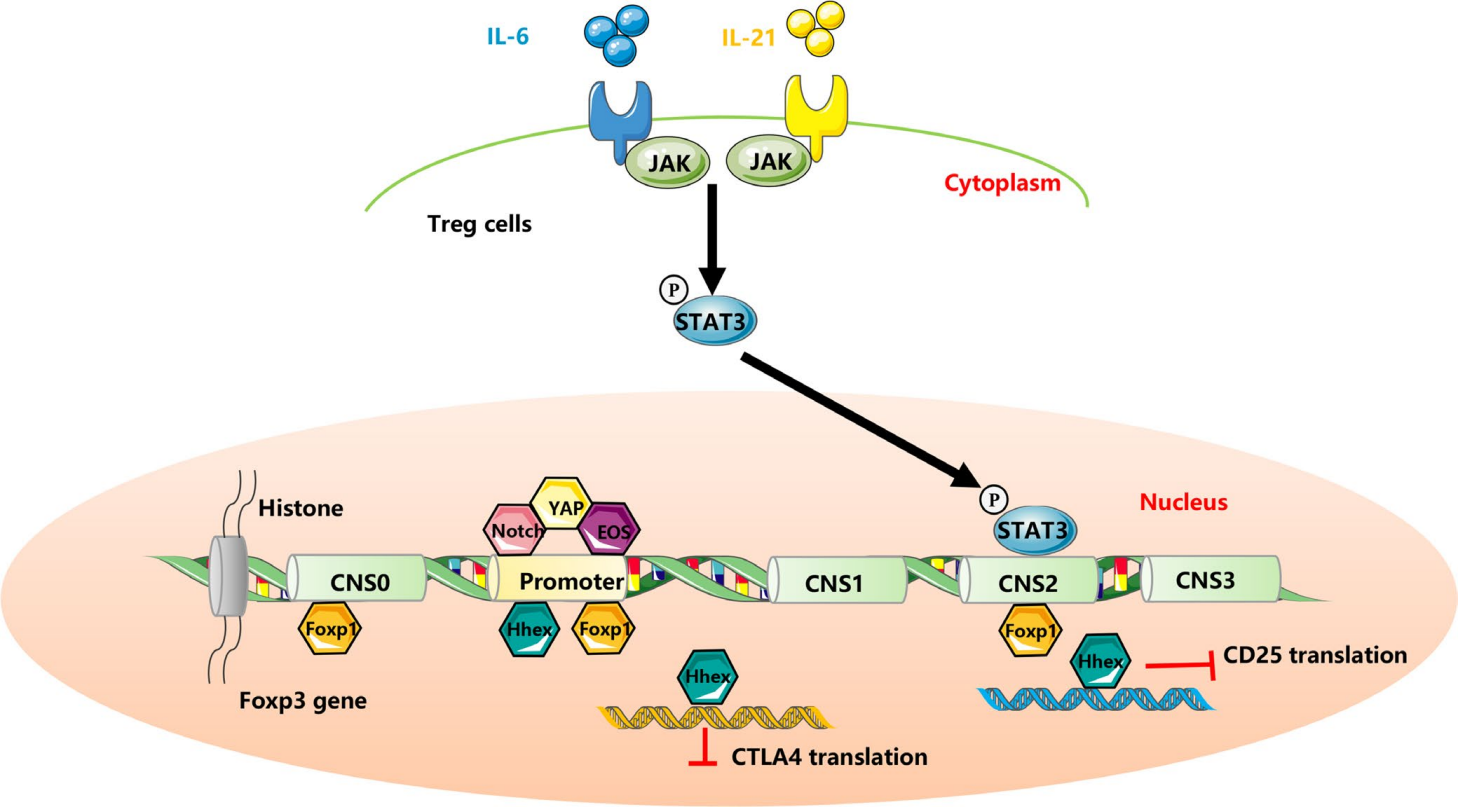
Inflammatory cytokines and transcription factors mediated regulation of Treg immunosuppression. IL‐6 and IL‐23 activate STAT3, which repress the transcription of Foxp3. Transcriptional factors regulate the suppressive function of Tregs through binding to promoter and conserved non‐coding sequences (CNS0, 1, 2, 3) at Foxp3 locus to regulate the transcription of Foxp3, or via regulating the transactivation of Treg signature genes CD25 and CTLA4

Multiple transcription factors have been shown to bind to the promoter of Foxp3 and act as positive or negative regulator of Foxp3 to modulate the stability, differentiation and suppressive function of Tregs (Figure 1).

Among the limited negative transcription factors of Foxp3, haematopoietically expressed homeobox (Hhex) is a negative regulator which inhibits the suppressive function of Tregs. In Treg‐potentiated differentiation condition which requires TGF‐β, Hhex expression is inhibited through a TGF‐β/Smad3 signalling pathway. Hhex binds to the promoter of Foxp3 and represses the transactivation of Treg signature genes including CTLA4 and IL2RA. Moreover, Hhex inhibited the suppressive functions of Tregs and failed to prevent colitis in the mouse model of inflammatory bowel disease (IBD).^16^

Foxp1, another member of the forkhead box (Foxp) subfamily, is identified to be a partner of Foxp3. Mice deficient in Foxp1 developed a multiorgan inflammatory disease. In DSS‐induced colitis and EAE mouse models, Foxp1‐deficient mice failed to display immunosuppressive functions and exaggerated the inflammatory responses. Foxp1 is further characterized to bind to the promoter, conserved non‐coding sequence (CNS)2 and CNS0 regions in Foxp3 locus. Moreover, Foxp1 and Foxp3 co‐ordinately bind to the promoter of CTLA‐4, a key ‘checkpoint’ molecule in maintaining the suppressive function of Tregs.^17^

Eos (Ikzf4), a transcription factor belongs to the Ikaros family, was recently shown to be important in maintaining the suppressive function of Tregs. Selective deletion of Eos in IBD and EAE mouse models displayed defective suppressive function of Tregs in vivo, with an observed disease progression and an increase of IL‐17 in CD4^+^ T cells. However, Tregs from EOS‐deficient mice endow the same suppressive function as the control mice *in vitro*.^18^ Another study using siRNA knockdown technology reported controversial results. Eos knockdown abrogated the suppressive function of Tregs both in vivo and *in vitro*.^19^

Other positive regulator of Treg suppressive function, such as YAP,^20^ has also been discovered. In summary, these transcription factors, either positively or negatively, function mainly through the following three mechanisms to regulate the function of Tregs. First, by binding to the Foxp3 locus, they are capable of regulating its expression. Secondly, they can act as partners of Foxp3 and physically bind to Foxp3 by protein‐protein interaction, which may regulate its structure and induce chromatin modifications. Thirdly, some of these transcription factors such as Hhex bind to the promoters of Treg signature genes such as CTLA4 and IL2RA (Figure 1).

### 2.3 Signalling pathways

Among the identified signalling pathways, NF‐κB shows its importance in maintaining Treg stability and suppressive function. NF‐κB signalling is classified into two pathways: the canonical pathway which encompasses p50, c‐Rel and p65 (RelA), and the non‐canonical pathway which involves TNF receptor family members (TNFR), NF‐κB‐inducing kinase (NIK), p100 and RelB.

The canonical signalling is proven to be the mast regulator of Treg development and for the maintenance of Treg suppressive function. In mice, selective deletion of Rela in Tregs lost their ability to suppress the proliferation of Teff cells. Moreover, the secretion of inflammatory cytokines IL‐17 and IFN‐γ was increased. Deletion of both Rela and c‐rel in Tregs failed to prevent the colitis of mouse models, which indicated that deletion of c‐rel and Rela led to complete loss of Treg suppressive function in vivo and in vitro.^21^ As constitutive TCR signalling is required for the maintenance of Treg suppressive function, it is reasonable that TCR signalling involved canonical activation of NF‐κB pathway is important for Treg function.

The alternative non‐canonical pathway of NF‐κB shows great potential in regulating the Treg function and homeostasis. Conditional deletion of p100 gene, NFκB2 in Tregs showed an increase in RelB activation and an expansion in Treg numbers. However, the suppressive function of Tregs is largely reduced compared to WT Tregs. In vivo, NFκB2‐deficient mice displayed a colonic inflammation after 12 months of age, accompanied with an increase of IFN‐γ and IL‐17 in Tregs.^22^ TNFR2, which is highly expressed in most Tregs, is crucial in maintaining the immune homeostasis and immunosuppressive properties of Tregs. Conditional ablation of TNFR2 in Foxp3^+^ cells reduced Treg suppressive function and diminished the expression of Tregs signature genes such as Foxp3, CD25 and CTLA‐4.^23^ IKKα, which is required for the non‐canonical activation of NF‐κB, is also required for the proliferative and suppressive function of Tregs. IKKα‐deficient Tregs are impaired in their proliferative and suppressive functions. In vivo, IKKα‐deficient Tregs failed to prevent the development of colitis.^24^ However, constitutive activation of NIK (NF‐κB‐inducing kinase), which links the TNFR to non‐canonical activation of NF‐κB, impaired the suppressive function of Tregs and endows Tregs with a pro‐inflammatory phenotype.^25^

Notch signalling, which is linked to canonical and non‐canonical activation of NF‐κB, is controversial in its role to regulate Treg activation and function. A few studies indicated the negative effect of Notch on Treg in RA, diabetes. In two arthritis mouse models, blockade of Notch1 increased the Treg population and suppressive ability.^26^ Other studies suggested Notch signalling as positive regulators of Treg function. In these studies, Notch activation recruits complex to bind to Foxp3 promoter and activate its expression.^27^ Thus, Notch controls Treg function and homeostasis in a context‐dependent manner.

Other signalling, such as PI3K and TLR signalling, is also shown to be important for the maintenance of Treg suppressive function.^28^, ^29^ However, despite the fact that all the aforementioned signalling pathways are necessary for maintaining Treg suppressive function, the subtle difference in the level of activation such as hyperactivation of the signalling pathways can antagonize the suppressive function of Tregs.

### 2.4 Post‐translational modification (PTM)

Foxp3 is the master regulator of Treg development of suppressive activation. Previous reviews have already summarized that Foxp3 could be modulated by ubiquitination, phosphorylation, O‐GlcNAcylation, acetylation, ubiquitylation and methylation.^30^ Here we focused on the recent findings concerning the role of ubiquitination in the regulation of Treg function.

**FIGURE 2:**
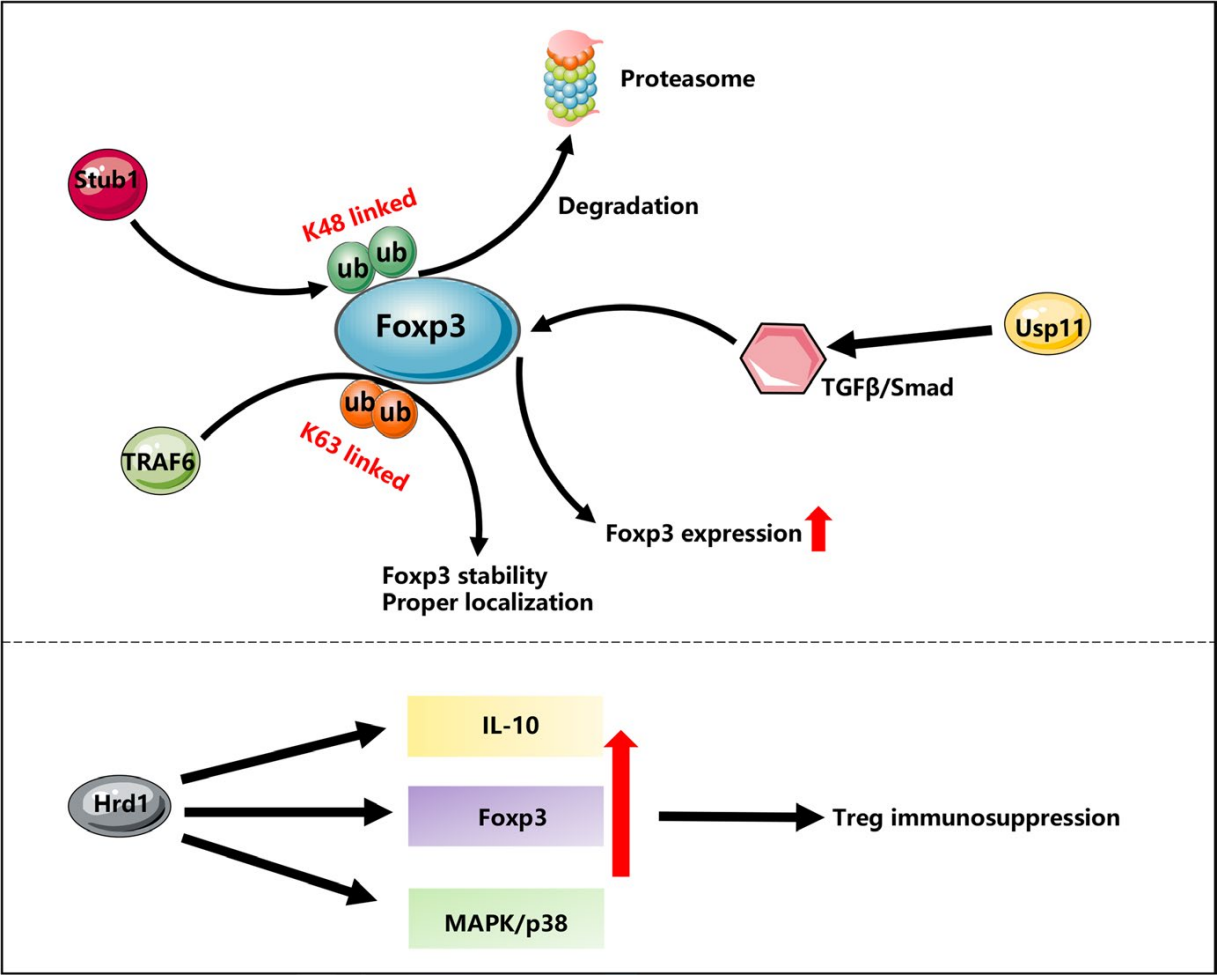
Ubiquitination mediated regulation of Treg immunosuppression. E3 ligase Stub1 regulates Foxp3 stability through K48‐linked ubiquitination, and E3 ligase TRAF6 regulates Foxp3 localization and localization through K63‐linked ubiquitination. Deubiquitinase Usp11 enhances Foxp3 expression through TGF‐β/Smad signalling. E3 ligase Hrd1 maintains the suppressive function of Tregs through up‐regulating the secretion of IL‐10, activating MAPK/p38 signalling and enhancing the expression of Foxp3

Although the best‐known function of ubiquitination is to direct proteins for degradation, it regulates diverse biological functions which depends both on specific targeted residue on substrates and the specific linkages types that formed between ubiquitin‐to‐ubiquitin. One mechanism by which ubiquitination modulates the suppressive function of Tregs is via regulating Foxp3 stability and localization. E3 ligase Stub1 promoted the degradation of Foxp3 via a K48‐linked ubiquitination. Overexpression of Stub1 in Tregs abrogated their suppressive function both in vivo and in vitro, which favoured their conversion into Th1‐like Tregs.^31^ Thus, Stub1 is negative regulator of Treg suppressive function. Recently, the same group characterized a dominant K63‐linked ubiquitination in the regulation of Treg function. Different from Stub1, E3 ligase TRAF6 directs a K63‐linked ubiquitination at lysine 262 of Foxp3, which ensures the proper localization of Foxp3. The absence of TRAF6 contributed to Foxp3 instability under treatment with pro‐inflammatory cytokines and led to Foxp3 perinuclear accumulation. Moreover, deletion of TRAF6 in Tregs and mutation of lysine 262 impaired the suppressive function of Tregs in vivo and in vitro. Taken together, TRAF6 is important in maintaining both the nuclear localization of Foxp3 and suppressive function of Tregs.^32^ Hrd1, an E3 ligase specifically located to the ER membrane, controls the ER stress response. Deletion of Hrd1 led to a decrease in IL‐10 secretion, a loss of Foxp3 and activation of ER stress–induced IRE1α and MAPK p38 activation, which contributed to loss of Treg suppressive functions in vivo.^33^ Besides, deubiquitinase Usp11 functions through activation of TGF‐β signalling to enhance Foxp3 expression and maintain the suppressive capacity of Tregs. Ectopic expression of USP11 led to enhanced suppressive function of Tregs in vivo and in vitro ^34^ (Figure 2).

### 2.5 miRNAs

miRNAs belong to the family of non‐coding RNAs, which regulate the expression of genes at post‐transcriptional level. Many genes implicated in regulation of Treg functions are potential targets of miRNAs; therefore, miRNA‐directed epigenetic modification is implicated in modulating the differentiation and the suppressive function of Tregs.

Most of these miRNAs are negative regulators of Treg suppressive function. However, miRNAs signal through multiple signalling pathways to exert their immunomodulatory functions. For example, overexpression of miR‐142‐3p in patients with granulomatosis with polyangiitis reduced the expression of target gene adenylate cyclase 9 (ADCY9), which is an enzyme that catalyses the conversion of ATP into cAMP. Thus, miR‐142‐3p reduced production of cAMP led to the impaired suppressive function of Tregs.^35^ miR‐142‐3p could also function through apoptotic pathways to regulate the suppressive function of induced Tregs (iTreg). In this case, knockdown of miR‐142‐3p up‐regulated the expression of anti‐apoptotic protein Bcl‐2 via targeting KDM6A‐H3K27me3 pathway, which enhanced the suppressive function of iTregs.^36^ Thus, miR‐142‐3p could signal through metabolic or histone modification‐directed epigenetic modification to inhibit the suppressive function of Tregs in a context‐dependent manner. In Graves disease, decreased level of miR‐23a‐3p signals through a SIRT1 involved demethylation of Foxp3 to impair the suppressive function of Tregs. Overexpression miR‐23a‐3p could restore the inhibitory function of Tregs both in vivo and in vitro.^37^ Furthermore, miR‐181a/b dampens the suppressive function of Tregs through down‐regulating the expression of Treg signature gene CTLA4. miR‐181a/b‐deficient mice displayed elevated suppressive capacity in vivo.^38^ Other mechanism, such as miR‐568 induced inhibition of NFAT5, a transcription factor, is also involved in inhibiting the suppressive function of Tregs.^39^

### 2.6 Protein and protein complex

Besides inflammatory cytokines, transcriptional factors, immunoreceptors and some protein complex are shown to regulate the functions of Tregs. The common pathways by which these proteins exert their immunomodulatory functions on the suppressive function of Tregs can be summarized in the following points: promotion or inhibition of inflammatory cytokines represented by IFN‐γ and IL‐17, modulation of Treg effector genes such as Klrg1, Icos, CD25, interference with the expression the Treg immune checkpoint molecules like CTLA4, GITR and OX40, modulation of Foxp3 stability and expression and activation or inactivation of signalling pathway related to Treg suppressive function.

**FIGURE 3:**
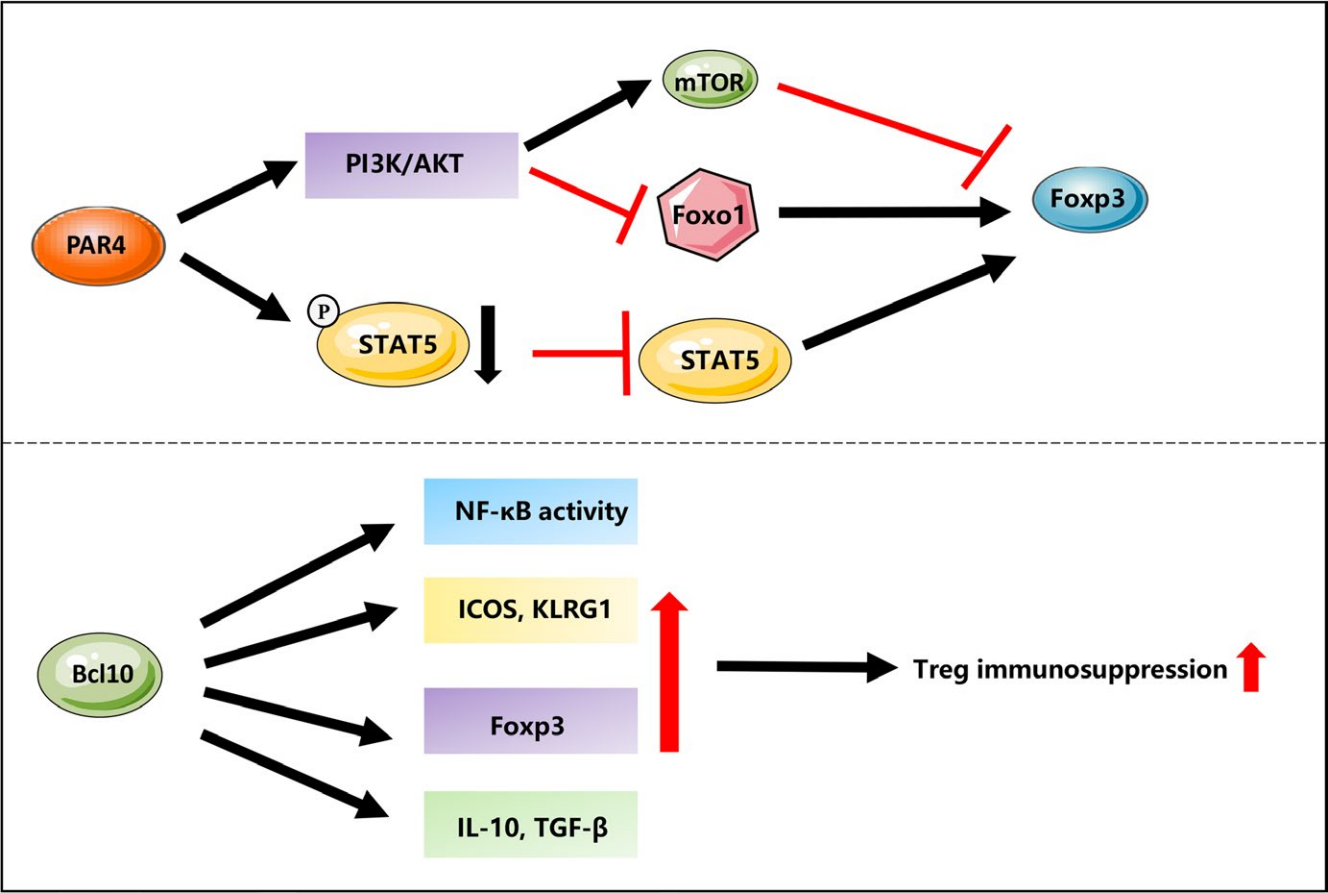
Protein and protein complex mediated regulation of Treg immunosuppression. PAR4 involved activation of PI3K‐AKT pathway to negatively regulate the transcription of Foxo1, and an inactivation of STAT5 pathway to co‐ordinate a negative regulation on Foxp3 expression. Scaffold protein Bcl10 promotes the activation of NF‐κB, the expression of Treg effector molecules such as KLRG1 and Icos, the master transcription factor Foxp3 and Treg‐associated suppressive molecules like TGF‐β1 and IL‐10 to maintain the suppressive function of Tregs

Among these proteins, protease‐activated receptor 4 (PAR4) is a negative regulator of Treg suppressive function. Despite its ubiquitous expression, PAR4 is dramatically increased during inflammation and infection. Tregs from PAR4 knockout mice displayed enhanced suppressive function of Tregs, which is consistent with the phenomenon observed from the WT Tregs treated with antagonist of PAR4. Analysis of the potential downstream pathway of PAR4 revealed a decreased phosphorylation of AKT‐Foxo1 and an enhanced phosphorylation of STAT5 and expression of PTEN in PAR4 knockout mice.^40^ This suggested a PAR4 involved activation of PI3K‐AKT‐Foxo1 pathway to negatively regulate the expression of PTEN and the stability, and an inactivation of STAT5 pathway to co‐ordinate a negative regulation on the suppressive function of Tregs (Figure 3).

Other protein complex or receptors, such as CoRest, neuropilin‐1 and Bcl10, are positive regulators of Treg functions. Bcl10 is a component of the scaffold protein CBM (Carma1‐Bcl10‐Malt1) to regulate the activation of NF‐κB and MAPK in T cells. Mice with specific deletion of Bcl10 in Tregs displayed an impaired the suppressive function of Tregs in vitro. Moreover, suppressive function of Bcl0‐deficient Tregs was decreased in an adoptive T cell transfer‐induced colitis model.^41^ Molecular mechanism analysis showed four potential pathways that Bcl10 might involve to maintain the suppressive function of Tregs: Knockdown of Bcl10 decreased the activation of NF‐κB, the expression of Treg effector molecules such as KLRG1 and Icos, the master transcription factor Foxp3 and Treg‐associated suppressive molecules like TGF‐β1 and IL‐10 (Figure 3).

Tregs exert immunosuppressive functions and prevent the development of autoimmune diseases. However, in tumour microenvironment, Tregs prevent anti‐cancer immunity by suppressing antitumour effector cells. Thus, in this specific tumour context, decreasing the suppressive function will favour antitumour therapies. Neuropilin‐1 (NRP‐1), a non‐tyrosine kinase receptor, is preferentially expressed in intratumoural Tregs. NRP1 interacts with semaphorin 4a and forms a complex with VEGFR2 to potentiate the Treg function and survival. Antagonist targeting NRP1 significantly reduced Foxp3 stability and enhanced IFN‐γ production, which dampened the suppressive function of intratumoural Tregs.^42^ Inhibition of NRP1 represented a prominent antitumour therapy as no systemic toxicity was observed in mouse models. The scaffolding protein CoREST, which is composed of the core element Rcor1, plus Hdac1/2 and Lsd1 enzymes, plays important roles in maintaining immunosuppressive function of Tregs and preventing antitumour immunity. Specific deletion of the core gene Rcor1 in mice led to decreased Treg suppressive function in vivo and in vitro, and a decrease of Hdac2 and Lsd1 enzymes. The decreased Hdac2 and Lsd1 increased the acetylation of histone3 at T‐bet promoter, which promote the expression of Th1‐signature cytokine IFN‐γ.^43^ While CoREST functions as a repressive factor, inhibition of CoREST enhanced the antitumour immunity mainly by an epigenetic mechanism.

### 2.7 Impact by other cells

Besides intrinsic factors, the phenotype and functions of Tregs can be influenced by extrinsic factors such as dendritic cells (DCs), natural killer cells (NKs) and mesenchymal stem cells (MSCs). These aforementioned cells impact Tregs via cell‐cell contact‐dependent manner or through secretory molecules like IL‐10, TGF‐β, IL‐2 and IL‐35. Moreover, these cells are able to convert the differentiation of Teff to Tregs.

Tregs were shown to interact with DC in tumours or autoimmunity. In these conditions, DCs are beneficial for the maintenance of Treg functionality. Depletion of DC in mice decreased the suppressive function of Treg to Teff in vivo, which led to intestinal inflammation in a mouse model of transfer colitis. Although Foxp3 expression is not changed in Tregs isolated from DC‐deleted mice, the higher expression of inhibitory receptors such as TIGHT and KLRG1 was noted.^44^ More recently, Niven et al^45^ focused on the autophagy in DCs in controlling the function of Tregs. Autophagy pathways are up‐regulated in DCs and are critical in controlling cell homeostasis. In mice with autophagy‐deficient Atg5 ^‐/‐^DCs, Tregs had a reduced suppressive function. Two molecular mechanisms might account for dysfunction of Tregs in autophagy‐deficient DC mice. On one side, higher expression of Hsp70 in Tregs from autophagy‐deficient mice might result in degradation of Foxp3 via ubiquitination‐dependent mechanism. On the other side, ICOS‐ligand, a molecule crucial for the stability and function of Tregs, is decreased in autophagy‐deficient DCs.

Other cells like MSCs have also shown immunomodulatory functions on Tregs. MSC induced in vivo the formation and expansion of Tregs.^46^ Post‐translational modifications like ubiquitination factors and epigenetic modification such as the hypomethylation of Treg‐specific demethylated region (TSDR) are required for the maintenance of Treg suppressive function. Khosravi et el.^47^ found that MSC‐induced Tregs displayed enhanced suppressive functions, which express high levels of E3 ligases TRAF6 and GRAIL and deubiquitination enzyme USP6. Moreover, Foxp3 TSDR demethylation was enhanced upon MSC stimulation. Thus, MSC signals through a ubiquitination and DNA demethylation‐dependent mechanism to induce the conversion and maintain the suppressive function of Tregs. Despite the MSCs and DCs which act as positive regulators of Tregs, NK cells act as a negative regulator of Treg function. In type 1 diabetes, NK cell is activated, which competed with Tregs for IL‐2, resulting in IL‐2 deprivation, Foxp3 down‐regulation and loss of Treg suppressive function.^48^

## 3 CONCLUSION

To summarize, we highlight the different factors which can influence the immunosuppressive functions of Tregs. Ranging from intrinsic factors like inflammatory cytokines, transcriptional factors, signalling pathways, surface molecules, protein complex, epigenetic modification and post‐translational modification, Tregs are subordinate to extrinsic factors such as the interaction with dendritic cells and mesenchymal stem cells. Given the evidence of context‐dependent function of Tregs, clarifying the specific factors in different disease condition is our future challenge in understanding the mechanism of dysfunctional Tregs.

Given the importance of Tregs in the aetiology of cancer and autoimmune disease, Treg‐targeted therapy has attracted much attention. Due to different roles of Tregs in cancer and autoimmune disease, the manipulation for up‐ or down‐regulation of Treg suppressive needs to differ. In autoimmune disease, Tregs exert protective function by suppressing the uncontrolled inflammation. In this context, the targeted therapy can be achieved by increasing the number of Treg cells or elevation of Treg suppressive function. The earliest approach is by adoptive transfer of Tregs. However, the side effects and the Treg instability limit the widespread application of this therapy.^49^ More recent trials for Treg immunotherapy have switched to IL‐2‐induced expansion of Tregs. As IL‐2 receptor is not specifically expressed in Tregs, IL‐2 stimulation can activate a range of IL‐2 responsive cells including CD4^+^ and CD8^+^ effector T cells and NK cells. Thus, the challenge is how to reduce the binding of IL‐2 to other non‐Treg cells. As Tregs expressed all three components of the IL‐2 receptor: αβγ, which differed from other cells expressing only IL‐2Rβγ, one optimal solution is to reduce IL‐2 affinity to beta‐gamma chain. For this, Peterson et al^50^ developed a long‐lived bivalent fusion protein IgG‐(IL‐2N88D)2, which showed reduced affinity for IL‐2Rβγ. Treatment of cynomolgus monkeys with low doses of this IL‐2 mutein protein allowed for a sustained 10‐ to 14‐fold increase in CD4^+^ and CD8^+^ Tregs, which opens up possibility for using IL‐2 therapy to treat autoimmune diseases. Multiple approaches that expand ex vivo Tregs in autoimmune disease are under investigation, but the carriers that could deliver these Tregs in vivo are the challenge. Nanoparticles (NPs) encapsulating peptides represent a potential modality to carry Tregs. It is reported that NPs encapsulating IL‐2 and TGF‐β coated with anti‐CD2/CD4 antibodies resulted in an expansion of Tregs in vivo and alleviated SLE phenotypes in mouse models.^51^

Although multiple approaches have been developed to expand Treg population, it is of vital importance to take consideration of Treg stability, Treg specificity and side effects into account to obtain an optimal approach for the treatment of autoimmune disease. In tumour microenvironment (TME), Tregs are a negative regulator as it suppresses antitumour immunity. In cancer, Treg‐targeted therapy is either to down‐regulate the suppressive function of Tregs or to deplete Treg population. Tregs in the TME express the IC molecules like CTLA‐4 and PD‐1, chemokines receptor like CCR4 and CCR8, and some proteins like Nrp1, can be utilized to deplete Tregs in TME. For example, anti‐PD‐1 therapy has been largely investigated in the treatment of cancer patients. Anti‐PD‐1 antibody is efficient in decreasing intratumoural Tregs and suppress tumour volume and tumour growth.^52^ Compared with traditional anti‐CTLA4 therapy, recently developed tumour‐conditional anti‐CTLA4 therapy depleted tumour‐infiltrated Tregs, while preserving tissue‐resident Tregs, which preserved antitumour effects and reduced multiorgan immune toxicity.^53^

To sum up, developing novel approaches to specifically target Tregs will represent next‐generation therapy for the treatment of autoimmune disease and cancers.

## CONFLICT OF INTERESTS

The authors declare no competing interests.

## AUTHOR CONTRIBUTIONS

L.Y: Writing of the manuscript and drafting of the figures. G. W and H.X: Edits and comments. All authors agreed on the final manuscript and figures.
